# Spatial distribution and determinants of alcohol consumption among pregnant women in Ethiopia: Spatial and multilevel analysis

**DOI:** 10.1371/journal.pone.0279405

**Published:** 2022-12-21

**Authors:** Tilahun Kassew, Bikis Liyew, Gebrekidan Ewnetu Tarekegn, Mesele Wondie, Tesfa Sewunet Alamneh, Sintayehu Asnakew, Shegaye Shumet

**Affiliations:** 1 Department of Psychiatry, School of Medicine, College of Medicine and Health Sciences, University of Gondar, Gondar, Ethiopia; 2 Department of Emergency Medicine and Critical Care Nursing, School of Nursing, College of Medicine and Health Sciences, University of Gondar, Gondar, Ethiopia; 3 Department of Epidemiology and Biostatistics, Institute of Public Health, College of Medicine and Health Sciences, University of Gondar, Gondar, Ethiopia; 4 Department of Psychiatry, School of Medicine, College of Health Science, Debre-Tabor University, Debre-Tabor, Ethiopia; Public Library of Science, UNITED STATES

## Abstract

**Background:**

Alcohol consumption during pregnancy is a known contributor to teratogen and causes a range of effects on pregnancy and birth outcomes. This study aimed to investigate the spatial variation and determinants of alcohol consumption among pregnant women in Ethiopia.

**Methods:**

A secondary data analysis was conducted using the 2016 Ethiopian Demographic and Health Survey data. A total of 1,135 pregnant women were included in the analysis. ArcGIS version 10.7 software was used to explore the spatial distribution of alcohol consumption, and SaTScan version 9.6 was employed to identify the significant spatial clusters of alcohol consumption. A mixed multi-level logistic regression analysis was employed to identify the determinant factors of alcohol consumption during pregnancy.

**Results:**

The result showed that the prevalence of alcohol consumption during pregnancy was 22.49% (with a 95% CI: 18.18 to 26.17). The spatial analysis showed that the spatial distribution of alcohol consumption significantly varied across the country [Global Moran’s I value = 0.30 (P<0.001)]. The SaTScan analysis identified two most likely clusters with high rates of alcohol consumption such as northwest Ethiopia (Log-Likelihood Ratio (LLR) = 155.56, p*<*0.001) and central Ethiopia (LLR = 19.27, p<0.01). Never in union, divorced and/ widowed [Adjusted odds ratio (AOR) = 2.56; 95% CI: *1*.*07*, *10*.*14*], attended primary school [AOR = 0.45; 95% CI: *0*.*27*, *0*.*95*], having two or more lifetime sexual partners [AOR = 2.59; 95% CI: *1*.*11*, *6*.*18*], living in rural [AOR = 1.52; 95% CI: *1*.*12*, *2*.*93*] and higher community media exposure [AOR = 0.54; 95% CI: *0*.*28*, *0*.*97*] were the factors associated with alcohol consumption.

**Conclusion:**

Alcohol consumption during pregnancy in Ethiopia was high. The spatial distribution of alcohol consumption was significantly varied across the country. Therefore, public health interventions targeting areas with high alcohol consumption are needed for drinking cessation and to prevent poor pregnancy outcomes related to alcohol use.

## Introduction

Pregnancy alcohol consumption is a modifiable risk factor for adverse effects on mothers and birth outcomes [1,2]. Maternal alcohol consumption is a known contributor to the high incidence of preterm delivery, low birth weight, spontaneous abortion, congenital malformations, and intrauterine growth retardation [3,4]. It results in a substantial risk of fetal alcohol spectrum disorders (FASDs) and fetal alcohol syndrome (FAS) [5,6]. FAS affects a newborn child’s development in a number of ways, including learning and memory impairments, attention deficits, and troubles with social and emotional development [3,5,7]. Consumption of alcohol during the first trimester is extremely hazardous to the health and neurodevelopment of the baby [8,9]. Negative pregnancy outcomes can be reduced by avoiding alcohol consumption because there is no safe amount of alcohol for pregnant women [10].

Globally, the estimated prevalence of alcohol consumption among pregnant women was 9.8% [11]. As the study report of different sub-Saharan African countries among pregnant women indicated, the prevalence of maternal alcohol consumption ranges from 2.5% to 59.3% [2,12–14], and from 3.4% to 20.5% in eastern Africa WHO region [11]. Alcohol consumption is widespread in Ethiopia, and it is used in a variety of social and religious contexts. As previous studies indicated, in Ethiopia, the prevalence of alcohol consumption among pregnant women was ranging from 7.9% to 34% [11,15,16]. These studies also showed the discrepancy in alcohol consumption across different geographical settings. Younger maternal age, divorce in marital status, lower educational level, unplanned pregnancy, lack of awareness about the effect of alcohol consumption, husband alcohol consumption, presence of partners and friends who consume alcohol, unemployment, and low socioeconomic status were the factors contributing for alcohol consumption among pregnant women [15,17–19]. Intimate partner violence, null parity, current tobacco smoking and use of other substances like Khat, cocaine, and marijuana during pregnancy, previous alcohol and other substance use when they were not pregnant, and having chronic medical and mental illness were also important predictors of maternal alcohol consumption [2,17,20,21]. It is also indicated that women who are living in rural areas are at high risk for drinking during pregnancy [19,22].

Previous primary studies have focused on alcohol drinking among pregnant women attending the facilities and lack focus on community-based using the population dataset at the national level [16,23]. However, there were no studies about the spatial distribution of alcohol consumption among pregnant women in Ethiopia. The current study was based on the recent Ethiopian Demographic and Health (EDHS) data that incorporates the entire nation with a large representative sample size. In addition, the study employed a multilevel logistic model to accommodate the hierarchical nature of the EDHS data. Identifying the spatial distribution and determinants of alcohol consumption during pregnancy is crucial for policymakers to evaluate programs; design interventions in major hot spot areas, and for planning maternal health care services related to alcohol consumption to have a healthy newborn baby. The results of this study could also be used for the delivery of strong health information and to bolster the existing strategies regarding maternal alcohol consumption. Therefore, the study aimed to investigate the spatial distribution and identify the individual and community level determinants of alcohol consumption among pregnant women in Ethiopia based on the EDHS 2016 data.

## Methods and materials

### Study setting, participants and procedures

The study used the Ethiopian Demographic and Health Survey (EDHS) data of Ethiopia. Ethiopia is classified into nine regional states, two administrative cities, 611 Woredas, and 15,000 Kebeles. Administratively each region is divided into zones and zones into Woredas which is the third administrative division of the country. Finally, at the fourth level, Woredas are further subdivided into Kebeles which are the lowest administrative unit [24]. A population-based cross-sectional study was employed in Ethiopia in which the data was extracted from the EDHS 2016 dataset; this was collected from January 18 to June 27, 2016. All pregnant women aged 15–49 years in the selected enumeration areas of the survey were considered the study participants.

The Ethiopian Demographic and Health Statistics 2016 survey used a two-stage stratified cluster sampling technique. The sampling frame was selected from the 2007 Population and Housing Census [24]. The regions were stratified into urban and rural, producing 21 strata. In each stratum, sample Enumeration Areas (EA) were selected independently in two stages by using proportional allocation and implicit stratification. In the first stage, a total of 645 EA (202 in urban areas and 443 in rural areas) were selected out of 84,915 EA. In the second stage of selection, a mean number of 28 households per cluster were systematically selected supported by the newly created household listing. EAs with “0” longitude and latitude data were dropped. Among 645 EAs two of them were not included initially in the DHS coordinate file. Of 643 EAs 487 were included in our analysis, the rest EAs were excluded due to dropped the zero GPS cells. Further detailed information about the sampling procedures and household selection has existed in the 2016 EDHS report [24]. For this study, the 2016 EDHS of the women dataset were used. A weighted sample of 1,135 pregnant women was used for the final analysis.

### Study variables

The outcome variable for this study was alcohol consumption responses from the two survey questions. The first question was “have you ever taken a drink that contains alcohol?” and the second: “during the last 30 days, how many days did you have a drink that contains alcohol?” Current alcohol consumption was defined as those pregnant women who drank daily or had drunk in the past 30 days that contain alcohol based on these questions. The individual and community-level variables were considered independent variables in the study. Individual-level parameters comprised age, marital status, maternal education level, household wealth index, individual’s media exposure, current employment, tobacco smoking, Khat chewing, pregnancy term, number of sex partners/ husbands, wanted pregnancy, parity, and religion. The household wealth index was a categorized variable by the DHS as poorest, poor, middle, rich, and richest, and we used it as is for analysis; we have used it as it is. Some of these factors were re-categorized for the simplicity of analysis. Only half of the pregnant women in the data were screened for intimate partner violence and partner/husband alcohol consumption, hence these parameters were not included in the analysis.

The variables considered as community-level factors were the place of residence and community-level media exposure. In the EDHS, participants’ media exposure was ascertained by 3 survey questions: “how often do you have read newspaper or magazine; how often do you listen to the radio, and how often do you have watching television? The responses were “not at all”, “at least once a week” and “more than once a week” for each question. Based on these questions, the individual level of media exposure was obtained by aggregating the specified ways of getting information such as reading news or magazine, listening to the radio, and watching television which gives a sum-total score ranging from zero to six. Then, the total score of media exposure was categorized as “yes” if the total score was greater than zero and “no” if the sum score was zero. Therefore, in this study, individuals’ media exposure was defined as those individuals who have a chance to get information through at least one of a specified mass media such as reading news or magazines, listening to the radio, and/or watching television at least once per a week.

The community-level media exposure was obtained by aggregating the individual-level media exposure into clusters by using the proportion of those who had media exposure. This community-level media exposure shows the general media exposure within the community. Since the aggregated variable had a skewed distribution, and therefore median values were used to categorize as higher and lower.

### Data management and statistical analysis

Data extraction, recoding, and descriptive statistics such as frequencies and percentages of variables were done using STATA-version 14. Sampling weights were performed before the analysis to restore the representativeness and to adjust the non-proportional allocation of the sample to strata and regions during the survey process. After the data adjustment and description, three statistical analysis models were preformed such as spatial autocorrelation and interpolation, spatial Scan distribution, and multilevel logistic regression analysis. Spatial distribution and a mixed multi-level logistic regression model were employed to identify the spatial variation and determinant factors of alcohol consumption during pregnancy, respectively.

#### Spatial data analysis

Spatial data analysis was performed using ArcGIS version 10.7 and Spatial Scan Statistics (SaTScanTM version 9.6) software. ArcGIS 10.7 was used for doing Moran’s I Analysis. Global Moran’s I statistics was used to determine the presence of spatial autocorrelation and whether alcohol consumption was dispersed, clustered, or randomly distributed across the country. Moran’s I value close to -1 indicated dispersed, Moran’s I value close to + 1 indicated clustered, or Moran’s I value was zero indicated randomly distributed. Moran’s I p-value < 0.05 indicated the presence of spatial autocorrelation. Hot spot analysis was done using Getis-Ord Gi* statistics to measure how spatial autocorrelation varies over the study location by calculating GI Bin for each area. High GIBin* in the statistical output indicated "hotspot" whereas low GI* indicated "cold spot. The ordinary Kriging spatial interpolation analysis was used to predict alcohol consumption for un-sampled areas based on sampled EAs.

**Spatial SaTscan analysis.** It was conducted using Kuldorff’s SaTscan version 9.6 software. This helps to identify the geographical locations of statistically significant spatial clusters of alcohol consumption among pregnant women. Pregnant women who were not drinking alcohol were considered controls, and those who were drinking alcohol were taken as cases represented by a 0/1 variable and fitted in the Bernoulli model. The number of cases in each location had a Bernoulli distribution and the model required data with or without alcohol consumption. The default maximum spatial cluster size of < 50% of the population was used as an upper limit, which allowed both small and large clusters to be detected and ignored clusters that contained more than the maximum limit. The result was reported using both table and figure. Areas with a high log likely hood ratio (LLR) and p-value < 0.05 were considered to high risk of alcohol consumption as compared to areas outside the window. Finally significant and most likely clusters with LLR, RR, and P-values were reported.

### Multi-level analysis

First bi-variable multilevel logistic regression analysis was performed using STATA-14 and those variables with a p-value *<*0.20 were selected for multivariable analysis. After selecting variables for multivariable analysis, four models; the null model (without explanatory variables), model II (containing only individual-level factors), model III (examined the effect of community-level factors), and model IV (which incorporates both individual and community level factors) were fitted. In the multivariable analysis, variables with a p-value of <0.05 were considered statistically significant and the factors associated with alcohol consumption were reported by an Adjusted Odds Ratio (AOR) at a 95% confidence interval. Model comparison and fitness was assessed using the log-likelihood and deviance and the model with a lower result of log-likelihood and deviance (Model IV) was considered the best–fitted model. The final model (model IV) was the best-fitted model and was selected for reporting of the results of the study. In addition, the measures of community variation (random effects) such as the Intra-Class Correlation (ICC), median odds ratio (MOR), and proportional change in variance (PCV) [25–27] were computed. These parameters were calculated to quantify; the degree of homogeneity of substance use within clusters, the degree of variation of substance use across clusters in terms of the odds ratio scale, and the proportion of variance explained by consecutive models, respectively.

### Ethics approval and consent to participate

Ethics approval was not required since this study is a secondary analysis based on the 2016 EDHS data. Before conducting our study, we registered and requested the dataset from DHS online archive and received approval to access and download the data files from the DHS website: https://dhsprogram.com/data/dataset_admin/index.cfm All DHS data should be treated as confidential, and no effort should be made to identify any household or individual respondent interviewed in the survey. The data could be used only for statistical reporting and analysis, and only for our registered research. According to the EDHS 2016 report, all respondents’ data were anonymized during the collection of the survey data [24].

## Results

### Socio-demographic, pregnancy, and behavioral characteristics

Weighted samples of 1,135 pregnant women were included in this analysis of the data. Of the participants, 333 (29.29%) were aged between 25 and 29 years, and 43.12% of them were Muslims. The majority of the participants (96.59) were married; more than half (53.20) had no formal education, and nearly three-quarters (73.93%) were working at a moment. Moreover, 85.83% of the women lived in rural areas, and 23.33% of the households had a lower wealth index. The majority (70.34%) of the women wanted the current pregnancy, and 448 (39.45%) of the women were in the second trimester of their pregnancy (**Table 1**).

**Table 1:** Socio-demographic, pregnancy and behavioral characteristics of pregnant women according to EDHS of 2016 (n = 1135).

Variables	Categories	Frequency (n)	Percent (%)
Age in years	15–19	101	8.93
	20–24	299	26.34
	25–29	333	29.29
	30–34	223	19.64
	35–39	126	11.11
	>40	53	4.69
Marital status	Married	1088	95.86
	Never in union	11	0.97
	Widowed	10	0.88
	Divorced	26	2.29
Religion	Orthodox	365	32.15
	Protestant	244	21.50
	Muslim	489	43.12
	Others*	37	3.23
Educational status	No formal education	604	53.20
	Primary school	397	35.03
	Secondary school	94	8.28
	Higher	40	3.49
Working status	Not working	839	73.93
	Yes working	296	26.07
Media exposure	No	726	63.97
	Yes	409	36.03
Household Wealth index	Poorest	257	22.67
	Poorer	265	23.33
	Middle	213	18.77
	Richer	198	17.45
	Richest	202	7.78
Region	Tigray	56	4.93
	Afar	12	1.07
	Amhara	220	19.41
	Oromia	496	41.93
	Somali	59	5.24
	Benishangul	11.65	1.03
	SNNP	264	23.26
	Gambella	3	0.23
	Harari	4	0.31
	Addis Ababa	24	2.16
	Dire Dawa	5	0.44
Living Residence	Urban	161	14.17
	Rural	974	85.83
Term of pregnancy	First trimester	241	21.23
	Second trimester	448	39.45
	Third trimester	446	39.32
Number of living children	No	240	21.19
	1–3 children	550	48.41
	4–6 children	284	25.06
	>6 children	61	5.33
Number of sex partners	One	908	79.96
	Two or more	227	20.04
Current pregnancy wanted	Yes	1,028	90.61
	Not at all	107	9.39
Ever terminated pregnancy	No	1,025	90.33
	Yes	110	9.67
Visited by health field workers	No	786	69.26
	Yes	349	30.74
Smoke tobacco products	No	1,120	98.72
	Yes	15	1.28
Ever chewed Khat	No	948	83.56
	Yes	187	16.44

Note:-

Others*: Catholic, traditional; Others**: Never in union, divorced, widowed, and/separated.

### Prevalence of alcohol consumption among the pregnant women

**Fig 1:**
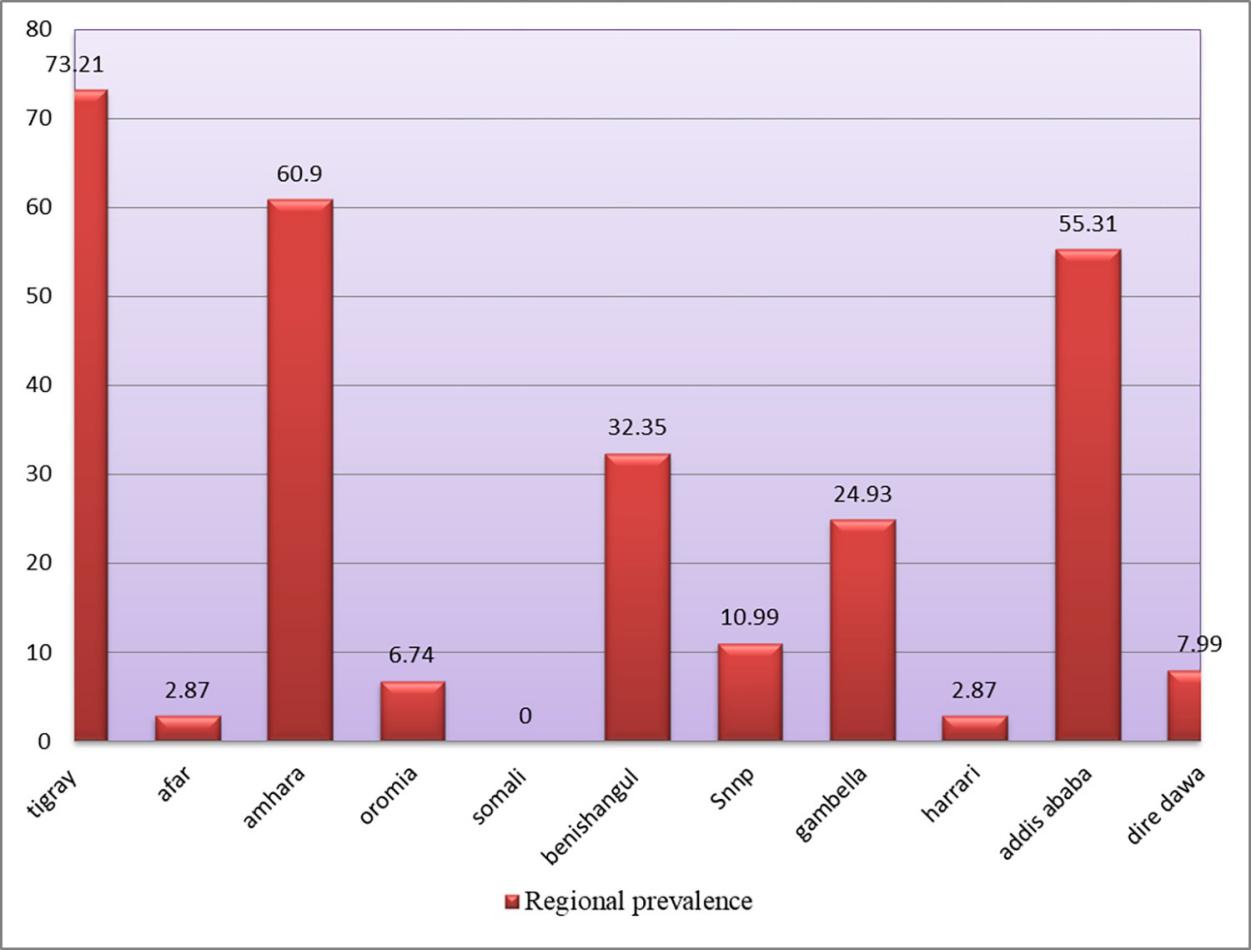
Regional prevalence of alcohol consumption during pregnancy in Ethiopia, 2016.

The estimated prevalence of alcohol consumption during the current pregnancy among pregnant women in Ethiopia was 22.49% with a 95% CI of 18.18 to 26.17. It is varied across the regions of the country. The highest prevalence (73.21%) was observed in the Tigray region and the lowest (0%) in Somali (**Fig 1**).

### Spatial analysis result

#### Spatial distribution of alcohol consumption among pregnant women

**Fig 2:**
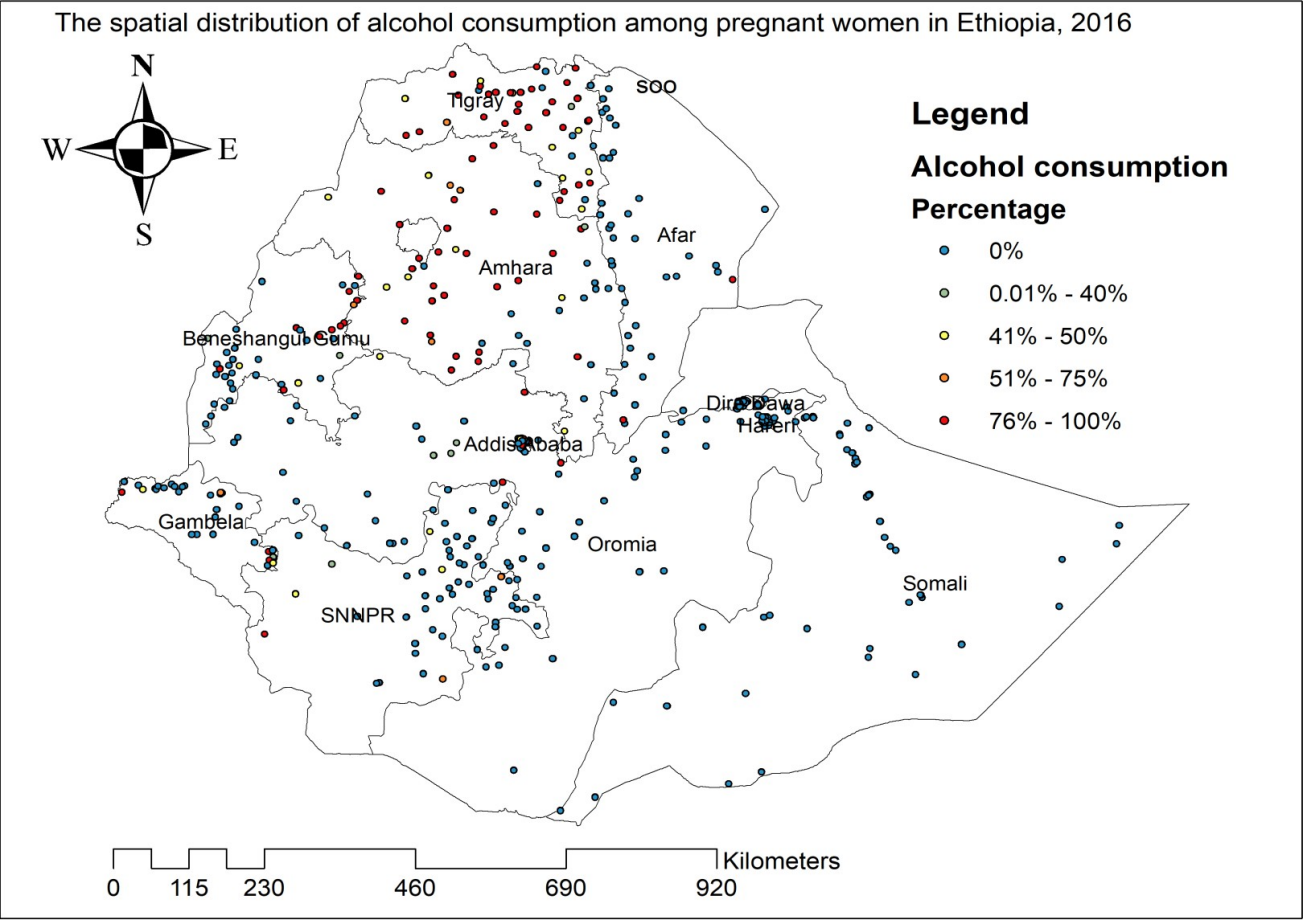
Spatial distribution of alcohol consumption among pregnant women across regions in Ethiopia, 2016.

As shown in **Fig 2**, the higher proportion of alcohol consumption has occurred in the entire part of Tigray, most parts of Amhara, Addis Ababa, and some parts of Benishangul Gumuz and Gambella regions. On the other hand, a low proportion of alcohol consumption occurred in Dire Dawa, Harari, Eastern Oromia, Afar, Somali, central Gambella, and Northeast SNNP regions of Ethiopia.

#### Spatial auto-correlation

**Fig 3:**
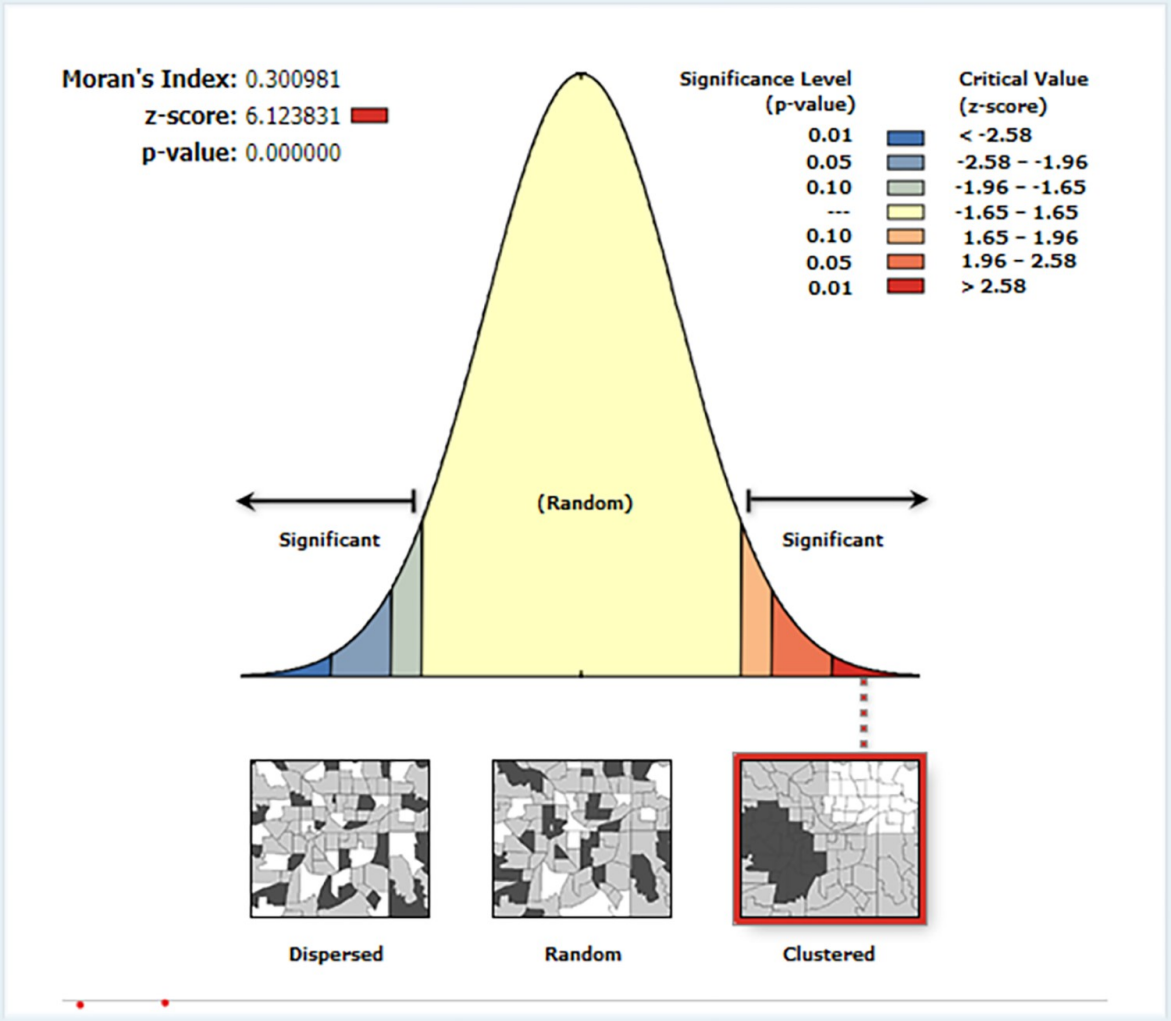
Spatial autocorrelation of alcohol consumption among pregnant women in Ethiopia, 2016 EDHS data.

In this study, the spatial distribution of alcohol consumption among pregnant women in Ethiopia was non-random with Global Moran’s I value = of 0.30 (P-value <0.001). High rates of alcohol consumption were observed across the country as shown in the clustered patterns (on the right side). A Z-score value of 6.124 indicated that there is less than a 1% likelihood that this clustered pattern could be the result of random chance (**Fig 3**).

### Hot spot analysis of alcohol consumption among pregnant women

**Fig 4:**
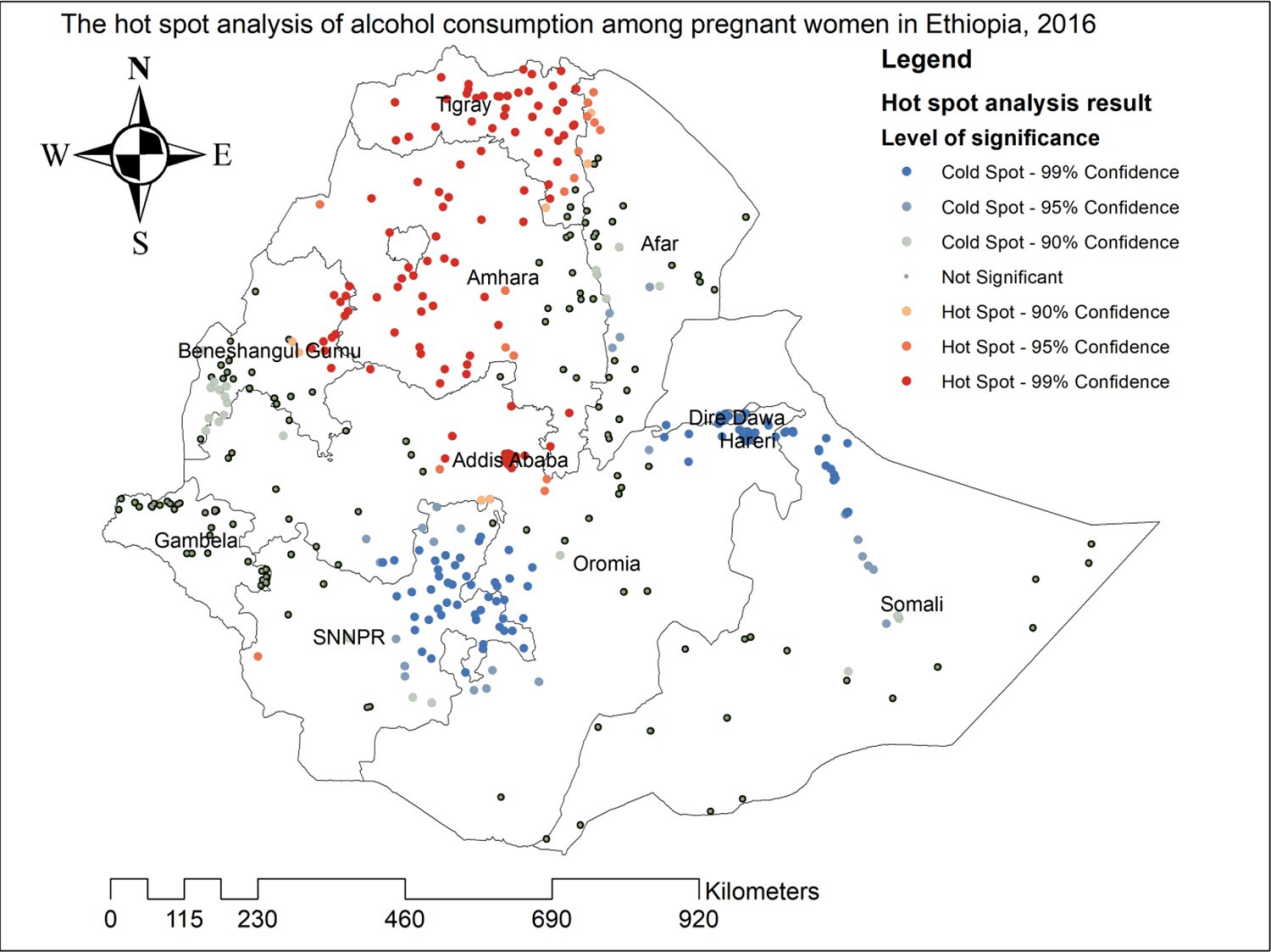
The Hot spot analysis of alcohol consumption among pregnant women in Ethiopia, 2016.

As shown in **Fig 4**, the red color indicated significant hot spot clusters (high alcohol consumption) and was observed in the entire part of Tigray, most parts of Amhara, Addis Ababa, east Beneshangul Gumuz, north Afar, and the northern part of the Oromia regions.

### Spatial interpolation of alcohol consumption

**Fig 5:**
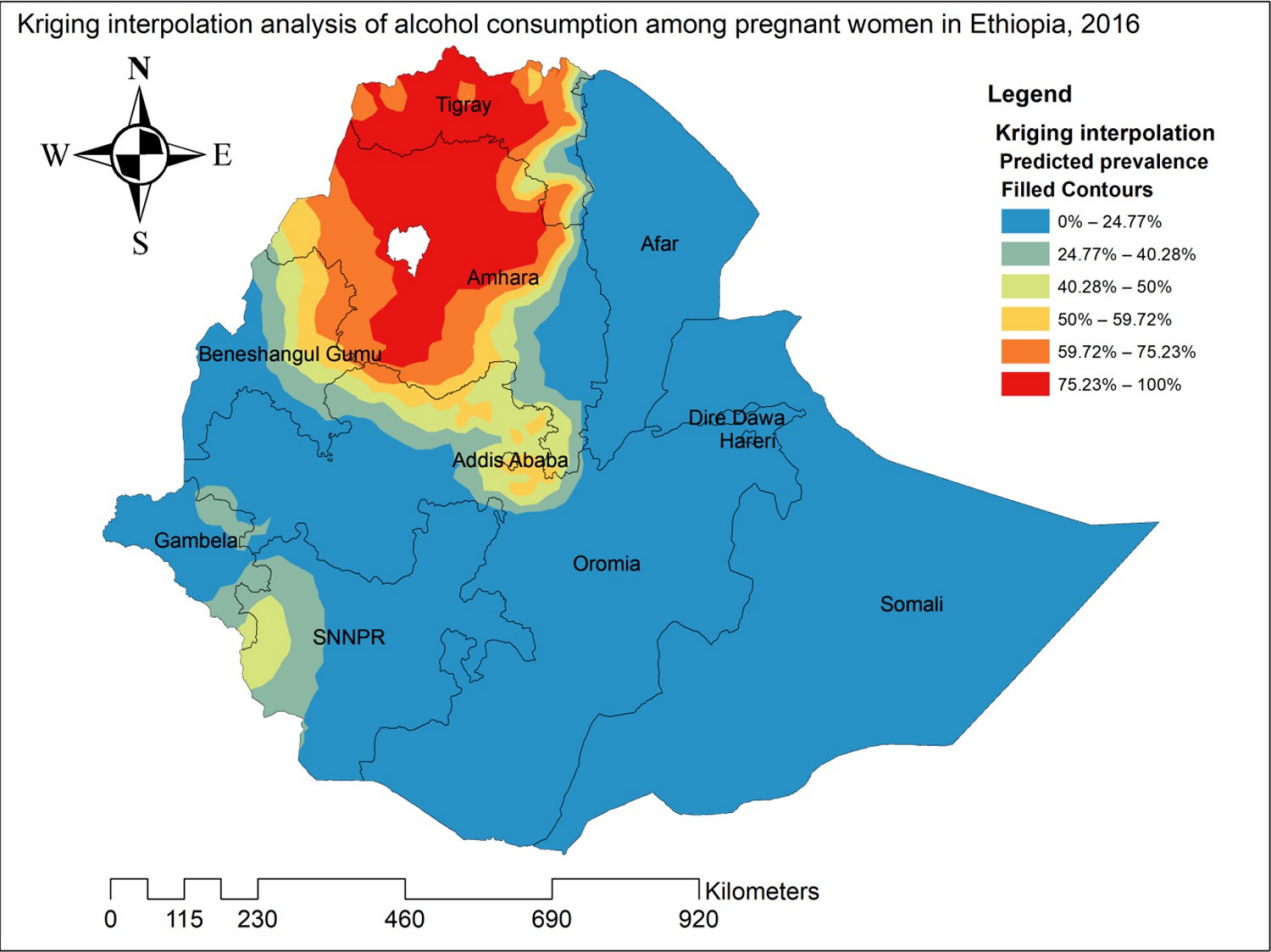
Spatial interpolation of alcohol consumption among pregnant women in Ethiopia, 2016.

Based on the ordinary Kriging interpolation analysis, the red color indicated the more intense areas of alcohol consumption as observed from the predicted prevalence-filled contours. The entire part of Tigray and the majority part of Amhara regions were identified as predicted more intense areas of alcohol consumption as compared to other regions of the country with a prevalence of 75.23% to 100% (**Fig 5**).

#### Spatial scan statistical analysis

**Fig 6:**
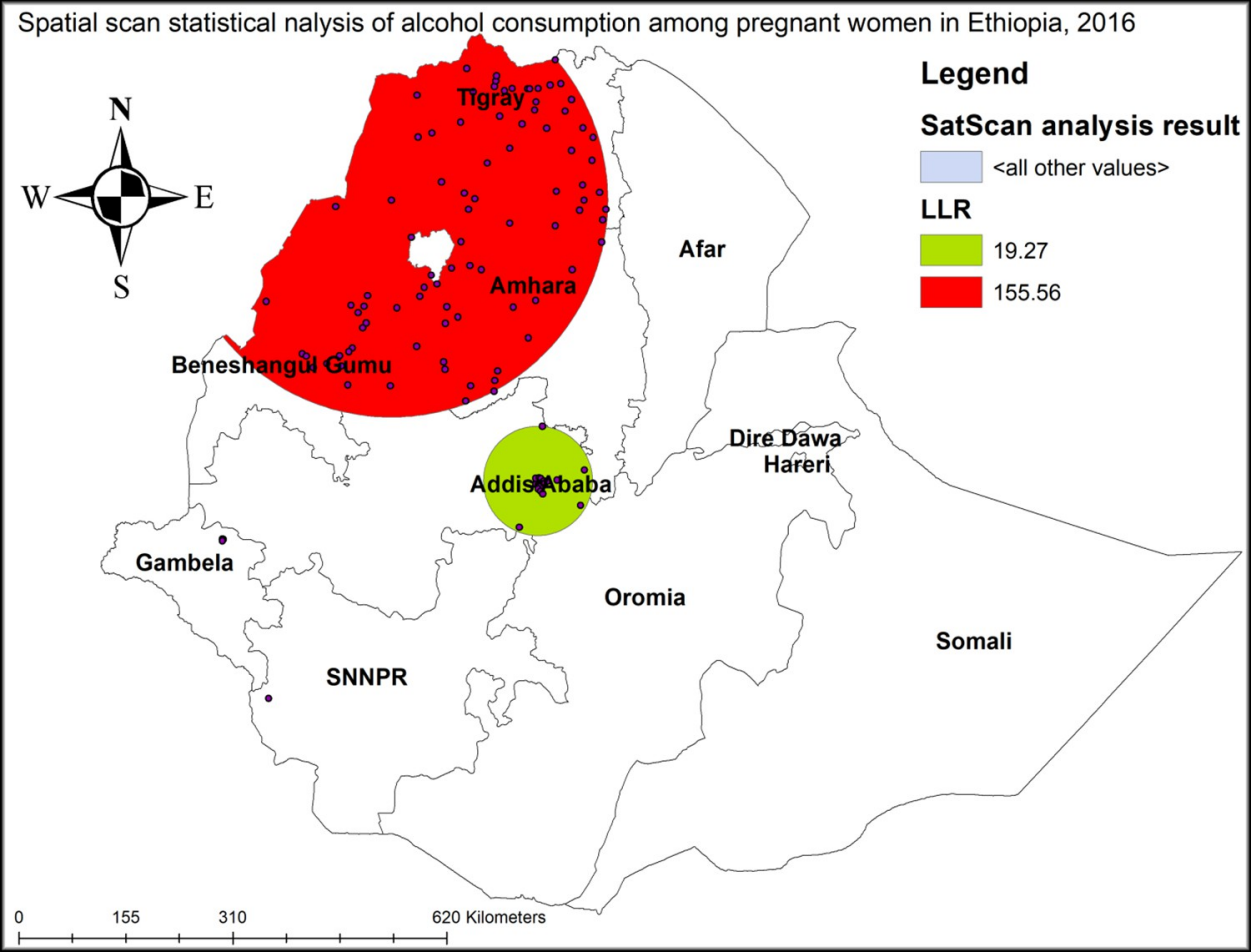
SaTScan analysis of hotspot clusters of alcohol consumption among pregnant women in Ethiopia, 2016.

A spatial scan statistical analysis identified 2 significant clusters with a total of 124 enumeration areas, of them the first was a primary (most likely) cluster, and the left one was a secondary cluster. The primary cluster spatial window red color as shown in **Fig 6** was located in the majority parts of the Tigray and Amhara regions that were centered at 12.669915 N, 36.775082 E with 313.13 km radius, a Relative Risk (RR) of 7.47 and Log-Likelihood Ratio (LLR) of 155.56, at p-value *<*0.001 (**Table 2**). It showed that the pregnant women inside the spatial window had 7.47 times higher risk of consuming alcohol than pregnant women outside the spatial window.

**Table 2:** SaTScan analysis result of alcohol consumption among pregnant women in Ethiopia, 2016.

Clusters	Enumeration areas	Coordinate/ radius	Population	Case	RR	LLR	P-value
1 (88)	279, 292, 638, 52, 296, 312, 327, 612, 163, 152, 169, 158, 431, 73, 602, 516, 512, 80, 258, 361, 253, 541, 386, 132, 548, 167, 199, 515, 425, 615, 429, 403, 583, 340, 3, 551, 268, 246, 98, 559, 181, 255, 256, 38, 528, 36, 584, 542, 66, 156, 375, 579, 150, 636, 183, 545, 597, 474, 35, 364, 400, 244, 81, 494, 538, 300, 184, 392, 136, 591, 176, 355, 84, 482, 531, 430, 160, 45, 604, 229, 449, 94, 351, 218, 200, 442, 479, 350	(12.669915 N, 36.775082 E) / 313.13 km	167	128	7.47	155.56	<0.001
2 (36)	108, 31, 11, 339, 414, 532, 582, 59, 463, 159, 144, 153, 274, 112, 293, 645, 464, 19, 110, 225, 61, 428, 451, 509, 539, 330, 211, 261, 402, 252, 353, 303, 121, 125, 217, 423	(9.03217N, 38.733008 E) / 78.91 km	56	32	3.09	19.27	<0.01

### Multilevel logistic regression analysis

#### Random effects and model fitness

There was a significant variation in log odds of alcohol consumption during pregnancy across the communities in the null model (Model-I) (σ2 u0 = 4.70, P < 0.001). This variation remained significant after controlling the individual- and community-level factors in all models. According to the computed ICC coefficient, differences in clusters account for about 58.83 percent of the overall variation in pregnant women’s alcohol consumption, while individual differences account for 41.27 percent. The null model also had the highest MOR value (7.84) indicating that when individuals were randomly selected from one cluster with a higher risk of alcohol consumption and the other cluster with a lower risk, those in the higher risk cluster had 7.84 times higher odds of alcohol consumption than those in the lower risk cluster. In addition, the highest (65.5%) PCV in the full model (model IV), indicates that the combined factors at both the individual and community levels explained 65.5 percent of the variation in log odds of having alcohol consumption variation across the community level (**Table 3**).

**Table 3:** Model comparison and goodness of fit test in the multilevel analysis.

Random effect	Model I	Model II	Model III	Model IV
Community variation	4.70 (.17)**	2.26 (0.25)**	3.14 (0.42)*	1.62 (0.26)**
ICC	0.5882	0.4072	0.4883	0.2769
MOR	7.84	4.17	5.38	3.35
PCV	Ref	0.52	0.33	0.66
Model fit statistics	Model I	Model II	Model III	Model IV
Log-likelihood ratio test	-393.25	-245.47	- 368.62	-226.26
Deviance	786.50	490.94	737.24	452.52

Note:

* = p-value <0.05

** = p-value<0.001.

Model fitness was determined using Log-likelihood and deviance values, as shown in Table 3, with lower values in the entire model (model IV), indicating that model-IV was a better explanatory model for alcohol consumption during pregnancy. This also suggests that the addition of community compositional factors increased the multilevel model’s ability, confirming the multilevel model’s goodness of fit.

#### The fixed effect analysis result

For the determinant factors analysis, the fixed-effect analysis was used. In multivariable multilevel logistic regression analysis, being never in the union, divorced and/ widowed, maternal education, having two or more lifetime sex partners, living in rural areas and higher community media exposure were all found to be significantly associated with alcohol consumption with a p-value of <0.05.

Mothers who were never married, divorced, or widowed in their marital status had 2.56 times [AOR = 2.56; 95% CI: *1*.*07*, *10*.*14*] higher odds of consuming alcohol while pregnant than those who were married. Women who had attended primary school education had 55% [AOR = 0.45; 95% CI: *0*.*27*, *0*.*95*] lower odds of alcohol consumption during the current pregnancy as compared to women who hadn’t attended formal education. The women who had two or more ever sex partners/ husbands had 2.59 times [AOR = 2.59; 95% CI: *1*.*11*, *6*.*18*] higher odds of alcohol consumption as compared with the women who had one sex partner/ husband. The odds of having alcohol consumption among women who are living in the rural areas was increased by 52% [AOR = 1.52; 95% CI: *1*.*12*, *2*.*93*] as compared to women living in urban. In terms of community media exposure, pregnant mothers who lived in a community with more media exposure had a 46% reduced odds of alcohol consumption [AOR = 0.54; 95% CI: *0*.*28*, *0*.*97*] than those who lived in a community with less media exposure (**Table 4**).

**Table 4:** Multivariable multilevel logistic regression analysis result of the individual and community level determinants of alcohol consumption among pregnant women in Ethiopia, 2016.

Variables	Model I	Model II (AOR 95% CI)	Model III(AOR 95% CI)	Model IV (AOR 95% CI)
Individual level factors				
Age in years 15–19 20–24 25–29 30–34 ≥35		1 0.56 (0.15, 1.90) 0.70 (0.20, 2.40) 0.56 (0.15, 2.01) 0.75 (0.19, 2.83)		1 0.53 (0.17, 1.71) 0.67 (0.21, 2.14) 0.66 (0.20, 2.21) 0.91 (0.25, 3.32)
Marital status Married Others		1 2.90 (1.66, 5.49)		1 *2*.*56 (1*.*07*,*10*.14)**
Maternal Educational No formal education Primary school Secondary school Higher		1 0.51 (0.32, 0.85) 0.77 (0.39, 1.52) 0.89 (0.25, 3.12)		1 *0*.*45 (0*.*27*, *0*.95)* 0.41 (0.13, 1.37) 0.74 (0.29, 1.60)
Currently working No Yes		1 0.94 (0.40, 2.21)		1 0.79 (0.37, 1.90)
Wealth Index Poorest Poorer Middle Richer Richest		1 1.06 (0.57, 1.98) 1.29 (0.65, 2.52) 0.90 (0.45, 1.81) 1.34 (0.63, 2.84)		1 0.92 (0.33, 2.51) 0.92 (0.34, 2.52) 0.40 (0.15, 1.05) 3.46 (0.57, 21.22)
Media exposure No Yes		1 1.32 (0.81, 2.18)		1 1.77 (0.86, 3.62)
Term of pregnancy First trimester Second trimester Third trimester		1 1.33 (0.72, 2.48) 1.36 (0.66, 2.54)		1 1.87 (0.79, 4.16) 2.01 (0.82, 4.86)
Sex partners One Two or more		1 2.71 (1.61, 4.58)		1 *2*.*59 (1*.*11*, *6*.18)**
Pregnancy wanted Yes No at all		1 1.37 (0.67, 2.84)		1 1.22 (0.37,4.09)
Ever chewed Khat No Yes		1 0.36 (0.17, 1.43)		1 0.27 (0.04, 1.07)
Community level variables				
Living Residence Urban Rural			1 2.04 (1.52, 3.06)	1 *1*.*52 (1*.*12*, *2*.93)*ab1
Community media exposure Lower Higher			1 0.85 (0.48, 1.51)	1 *0*.*54 (0*.*28*, *0*.97)*
Constant	0.26 (0.09,0.77)	0.14 (0.09, 0.23)	0.15 (0.05, 0.91)	0.08 (0.01, 0.60)

Note:

* = p-value<0.05

** = p-value<0.01; Others = never in union, divorced and/widowed.

## Discussion

Using nationally representative EDHS data, this study looked into the spatial distribution and determinants of alcohol intake among pregnant women in Ethiopia. The information presented was gathered before the COVID-19 epidemic. Alcohol consumption was found to be 22.49 percent in the current study. This finding is consistent with previous research in Uganda [19] and Congo [28]. This finding matched the findings of systematic review of research conducted in Sub-Saharan African countries [2]. This finding was lower than the results of previous studies done in northwest Ethiopia [15], South Africa [20], Nigeria [13], Ghana [29], and Russia [7]. It could be related to differences in study settings, screening instruments for alcohol consumption, and study design. Participants in the Ethiopian and Ghanaian studies, for example, were recruited from a small geographically located health facility. In the South Africa, Nigeria, and Russia studies, a standardized alcohol consumption identification tool questionnaire was employed. The current study, on the other hand, used national-level population-based data and only two alcohol use assessment DHS items. The Ghana study utilized a longitudinal study design, whereas this study employed a cross-sectional study design. A longitudinal follow-up of alcohol use patterns is more accurate than a one-time measurement of alcohol consumption status [30], and drinking behavior may be under-reported in the current cross-sectional study. Discrepancies in pregnant women’s drinking habits could be associated with social, cultural, religious, and health policy differences [31,32]. Furthermore, due to social desirability bias, recall bias, and societal and cultural norms regarding drinking alcohol, under-reporting of drinking behavior is common, making it difficult to determine the accurate magnitude and amount of alcohol consumption during pregnancy [2,33].

The result of this study, on the other hand, was higher than those of previous studies in central Ethiopia [34] and southern Ethiopia [16], Tanzania [14], Burkina Faso [35], and Sweden [32]. The reason for the discrepancy might be that the majority of studies mentioned are facility-based and have small sample sizes. This could also be related to the increased drinking pattern and availability of local alcoholic beverages such as “Tella”, “Areki” and “Tej” in the rural areas and agrarian regions of Ethiopia [36]. Furthermore, the discrepancy between this finding and those obtained outside of Ethiopia could be related to socio-demographic and cultural differences. The current study has shown many pregnant mothers and their fetuses in Ethiopia are suffering from the detrimental health impacts of alcohol consumption during pregnancy. As a result, an effective maternal health promotion program aimed at preventing alcohol-exposed pregnancy is essential, as is an understanding of why women drink during pregnancy.

The spatial analysis showed that the spatial distribution of alcohol consumption was highly variable across the country. In Tigray, Addis Ababa, Northern Afar, and the majority of Amhara and North Oromia regions, significant hotspot areas with high alcohol consumption were observed. The Christian religion may be the dominant religion in these areas, where consuming alcoholic beverages is socially accepted [37]. In addition, these areas are more agrarian regions where the survivors have produced local alcoholic beverages such as “Tella”, “Areki” and “Tej” in the rural areas, and “beer” and “wine” in the urban areas from agricultural products [36]. This finding suggests that policymakers and programmers should boost community-based maternal health interventions in order to improve the quality of treatment and positive mother and newborn health outcomes in these high-alcohol-consumption areas.

This study showed that alcohol consumption during pregnancy was significantly associated with marital status, education status, the number of sex partners/ husbands, residence, and community media exposure. Maternal alcohol consumption was significantly associated with unmarried, divorced and/or widowed marital status. This finding was consistent with those of previous studies [13,19,38]. These mothers who were not married, divorced, and/or widowed might live alone which leads to feelings of loneliness, helplessness, and perceive themselves as disadvantaged. Individuals with feelings of loneliness and negative emotions drink alcohol to cope with and escape from negative feelings. Maternal education level is an important contributor to alcohol use during pregnancy. The odd of alcohol consumption was lower among mothers who attended primary school compared to those who hadn’t attended formal education. This finding was supported by previous studies in Ethiopia [15], Nigeria [13], and the Netherlands [39]. This could be because women’s awareness of the potentially negative effects of alcohol on the fetus increases in tandem with their maternal education level. This forces the mother to limit and avoid using alcohol while pregnant.

We observed a significant association between maternal alcohol use during pregnancy and having two or more lifetime sexual partners in our study. Previous studies also support our finding [2,40], these studies have found a strong association between maternal alcohol use and multiple lifetime sexual partners. Unsuccessful previous sexual partner is the main reason to have more than one sexual partner which is associated with a woman to have social and psychological impacts. As a result, pregnant women might drink alcohol to get relief from their stress [41]. In addition, if a woman has a partner who drinks alcohol that can have the potential to force the woman to initiate and drink alcohol concordantly because different persons have different behaviors.

Fourth, the place of residence is associated with maternal alcohol use during pregnancy. Pregnant mothers from rural areas were more likely to consume alcohol than those in urban areas. This result is consistent with the studies done in Ethiopia [38], Uganda [19], and South Africa [22]. This might be due to the ease with which homemade alcoholic beverages such as “Tella”, “Areki” and “Tej” are produced and distributed in Ethiopia’s rural rather than urban areas. In this study, the majority of the participants were from rural areas where there is insufficient information sharing, discussion, and service delivery on the adverse effects of alcohol use during pregnancy. And it affects a large segment of the community that could pose challenges for intervention [38,42].

Furthermore, according to the current study, pregnant women who had higher media exposure at the community level were less likely drinks alcohol during pregnancy than those who had lived in a community with lower media exposure. This result is consistent with earlier findings [43,44]. This could be due to the presence of higher media exposure in the community helps to have media campaigns on alcohol consumption and its negative health-related outcomes [45]. Sharing information about alcohol and health with communities through mass media (reading newsletters/magazines, listening to the radio, and/or watching television) influences mothers to limit their alcohol consumption during pregnancy in order to maintain good health for themselves and their fetuses. This implies that expanding mass media campaigns and health education may be an effective way to increase mothers’ awareness of the dangerous effects of alcohol use during pregnancy.

### Strength and limitations of the study

The main strength of this study, it can be generalized to all women who were pregnant during the study period since the study was based on weighted data to make it representative at national and regional levels. Furthermore, the study used GIS and SaTScan statistical tests, and advanced analysis models. Those are crucial to detect significant hotspot areas of alcohol consumption and to get reliable standard errors and estimates. On the other hand, there are limitations to this work. One of the main limitations of this work might be the recall bias because the data collection took place preceding five years of the survey of the study. Under-reporting of drinking behavior is common because of social desirability bias regarding drinking alcohol which makes it difficult to determine the accurate magnitude and amount of alcohol consumption during pregnancy. Secondly, questions relating to alcohol consumption in the survey were not a standardized tool. Besides, the research could not show the cause-effect relationships between factors and outcomes owing to its cross-sectional nature.

### Conclusion

Alcohol consumption was prevalent among Ethiopian pregnant women. The spatial distribution of alcohol consumption was significantly varied across the country. Significant hotspot clusters with a high prevalence of alcohol consumption were identified in the entire part of Tigray, Addis Ababa, Northern Afar, majority parts of Amhara and North Oromia regions. Therefore, public health interventions targeting major hot spot clusters are needed for drinking cessation and to prevent poor pregnancy outcomes related to alcohol use. A Great effort of mass media campaigns should be done to enhance awareness of women about the adverse effect of alcohol intake during pregnancy, especially in rural areas. Again, early interventions target women at risk such as never being in a union, being divorced, and/or widowed, and those who have two or more lifetime sexual partners are important to achieve abstinence through support and assistance.

## Supporting information

S1 File (PDF)

## References

[1] Asamoah BO, Agardh A: Alcohol consumption in relation to maternal deaths from induced-abortions in Ghana. *Reproductive health* 2012, 9(1):1–9. 22867435 10.1186/1742-4755-9-10 PMC3453516

[2] Addila AE, Bisetegn TA, Gete YK, Mengistu MY, Beyene GM: Alcohol consumption and its associated factors among pregnant women in Sub-Saharan Africa: a systematic review and meta-analysis’ as given in the submission system. *Substance* Abuse Treatment, Prevention, and Policy 2020, 15:1–14. 32293479 10.1186/s13011-020-00269-3 PMC7158038

[3] Henriksen TB, Hjollund NH, Jensen TK, Bonde JP, Andersson A-M, Kolstad H, et al: Alcohol consumption at the time of conception and spontaneous abortion. *American Journal of Epidemiology* 2004, 160(7):661–667. 15383410 10.1093/aje/kwh259

[4] Ornoy A, Ergaz Z: Alcohol abuse in pregnant women: effects on the fetus and newborn, mode of action and maternal treatment. *International Journal of Environmental Research and Public Health* 2010, 7(2):364–379. 20616979 10.3390/ijerph7020364 PMC2872283

[5] Nayak RB, Murthy P: Fetal alcohol spectrum disorder. *Indian pediatrics* 2008, 45(12):977. 19129565

[6] Comasco E, Hallberg G, Helander A, Oreland L, Sundelin‐Wahlsten V: Alcohol consumption among pregnant women in a Swedish sample and its effects on the newborn outcomes. *Alcoholism*: *Clinical and Experimental Research* 2012, 36(10):1779–1786. 10.1111/j.1530-0277.2012.01783.x 22486280

[7] Balachova T, Bonner B, Chaffin M, Bard D, Isurina G, Tsvetkova L, et al: Women’s alcohol consumption and risk for alcohol‐exposed pregnancies in Russia. *Addiction* 2012, 107(1):109–117. 21752144 10.1111/j.1360-0443.2011.03569.x PMC3229961

[8] Nykjaer C, Alwan NA, Greenwood DC, Simpson NA, Hay AW, White KL, et al: Maternal alcohol intake prior to and during pregnancy and risk of adverse birth outcomes: evidence from a British cohort. *Journal of Epidemiol Community Health* 2014, 68(6):542–549. doi: 10.1136/jech-2013-202934 24616351 PMC4033207

[9] Colvin L, Payne J, Parsons D, Kurinczuk JJ, Bower C: Alcohol consumption during pregnancy in nonindigenous west Australian women. *Alcoholism*: *Clinical and Experimental Research* 2007, 31(2):276–284. 17250620 10.1111/j.1530-0277.2006.00303.x

[10] Isaksen AB, Østbye T, Mmbaga BT, Daltveit AK: Alcohol consumption among pregnant women in Northern Tanzania 2000–2010: a registry-based study. *BMC pregnancy and childbirth* 2015, 15(1):205. doi: 10.1186/s12884-015-0630-0 26337194 PMC4559883

[11] Popova S, Lange S, Probst C, Shield K, Kraicer‐Melamed H, Ferreira‐Borges C, et al: Actual and predicted prevalence of alcohol consumption during pregnancy in the WHO African Region. *Tropical Medicine & International Health* 2016, 21(10):1209–1239. 27429168 10.1111/tmi.12755

[12] Abasiubong F, Bassey EA, Udobang JA, Akinbami OS, Udoh SB, Idung AU: Self-Medication: potential risks and hazards among pregnant women in Uyo, Nigeria. *Pan African Medical Journal* 2012, 13(1). 23308320 PMC3527026

[13] Ordinioha B, Brisibe S: Alcohol consumption among pregnant women attending the ante. Natal clinic of a tertiary hospital in south. South Nigeria. *Nigerian journal of clinical practice* 2015, 18(1):13–17. 25511337 10.4103/1119-3077.146966

[14] Mpelo M, Kibusi SM, Moshi F, Nyundo A, Ntwenya JE, Mpondo BC: Prevalence and factors influencing alcohol use in pregnancy among women attending antenatal care in Dodoma region, Tanzania: a cross-sectional study. *Journal of pregnancy* 2018, 2018. doi: 10.1155/2018/8580318 30420920 PMC6211147

[15] Anteab K, Demtsu B, Megra M: Assessment of prevalence and associated factors of alcohol use during pregnancy among the dwellers of Bahir-Dar City, Northwest Ethiopia, 2014. 2014.

[16] Mekuriaw B, Belayneh Z, Shemelise T, Hussen R: Alcohol use and associated factors among women attending antenatal care in Southern Ethiopia: a facility based cross sectional study. *BMC research notes* 2019, 12(1):690. doi: 10.1186/s13104-019-4703-4 31651365 PMC6813970

[17] Dupraz J, Graff V, Barasche J, Etter J-F, Boulvain M: Tobacco and alcohol during pregnancy: prevalence and determinants in Geneva in 2008. *Swiss medical weekly* 2013, 143(2122). doi: 10.4414/smw.2013.13795 23740290

[18] Smith L, Savory J, Couves J, Burns E: Alcohol consumption during pregnancy: cross-sectional survey. *Midwifery* 2014, 30(12):1173–1178. doi: 10.1016/j.midw.2014.04.002 24815567

[19] Namagembe I, Jackson LW, Zullo MD, Frank SH, Byamugisha JK, Sethi AK: Consumption of alcoholic beverages among pregnant urban Ugandan women. *Maternal and child health journal* 2010, 14(4):492–500. 19629663 10.1007/s10995-009-0500-3 PMC2906221

[20] Hartley M, Tomlinson M, Greco E, Comulada WS, Stewart J, Le Roux I, et al: Depressed mood in pregnancy: prevalence and correlates in two Cape Town peri-urban settlements. *Reproductive health* 2011, 8(1):9. doi: 10.1186/1742-4755-8-9 21535876 PMC3113332

[21] Cui Y, Shooshtari S, Forget EL, Clara I, Cheung KF: Smoking during pregnancy: findings from the 2009–2010 Canadian Community Health Survey. *PloS one* 2014, 9(1):e84640. doi: 10.1371/journal.pone.0084640 24416257 PMC3885577

[22] Morojele NK, London L, Olorunju SA, Matjila MJ, Davids AS, Rendall-Mkosi KM: Predictors of risk of alcohol-exposed pregnancies among women in an urban and a rural area of South Africa. *Social Science & Medicine* 2010, 70(4):534–542. 19932549 10.1016/j.socscimed.2009.10.040

[23] Alamneh AA, Endris BS, Gebreyesus SH: Caffeine, alcohol, khat, and tobacco use during pregnancy in Butajira, South Central Ethiopia. *PloS one* 2020, 15(5):e0232712. doi: 10.1371/journal.pone.0232712 32384102 PMC7209255

[24] Agency CS: Ethiopia Demographic and Health Survey 2016. Key Indicators Report In. Addis Ababa, Ethiopia, and Rockville, Maryland, USA CSA and ICF; 2016.

[25] Weinmayr G, Dreyhaupt J, Jaensch A, Forastiere F, Strachan DP: Multilevel regression modelling to investigate variation in disease prevalence across locations. *International Journal of Epidemiology* 2017, 46(1):336–347. 27864412 10.1093/ije/dyw274

[26] Austin PC, Merlo J: Intermediate and advanced topics in multilevel logistic regression analysis. *Statistics in medicine* 2017, 36(20):3257–3277. doi: 10.1002/sim.7336 28543517 PMC5575471

[27] Merlo J, Chaix B, Ohlsson H, Beckman A, Johnell K, Hjerpe P, et al: A brief conceptual tutorial of multilevel analysis in social epidemiology: using measures of clustering in multilevel logistic regression to investigate contextual phenomena. *Journal of Epidemiology & Community Health* 2006, 60(4):290–297. 16537344 10.1136/jech.2004.029454 PMC2566165

[28] Moise IK: Alcohol use, pregnancy and associated risk factors: a pilot cross-sectional study of pregnant women attending prenatal care in an urban city. *BMC pregnancy and childbirth* 2019, 19(1):472. doi: 10.1186/s12884-019-2652-5 31805891 PMC6896278

[29] Lekettey JDP, Dako-Gyeke P, Agyemang SA, Aikins M: Alcohol consumption among pregnant women in James town community, Accra, Ghana. *Reproductive Health* 2017, 14(1):1–8. 28950877 10.1186/s12978-017-0384-4 PMC5615456

[30] Gil AG, Wagner EF, Vega WA: Acculturation, familism, and alcohol use among Latino adolescent males: Longitudinal relations. *Journal of Community Psychology* 2000, 28(4):443–458.

[31] Devaux M: Social disparities in alcohol drinking. *Tackling harmful alcohol use*: *economics and public health policy Paris*: *OECD Publishing* 2015:61–80.

[32] Skagerström J, Häggström-Nordin E, Alehagen S: The voice of non-pregnant women on alcohol consumption during pregnancy: a focus group study among women in Sweden. *BMC public health* 2015, 15(1):1–9. doi: 10.1186/s12889-015-2519-2 26621365 PMC4666188

[33] Colombo R, Preve M, Bolla E, Traber R: Seasonal variation and alcohol consumption: a retrospective observational study. *European Psychiatry* 2017, 41(S1):s874–s874.

[34] Wubetu AD, Habte S, Dagne K: Prevalence of risky alcohol use behavior and associated factors in pregnant antenatal care attendees in Debre Berhan, Ethiopia, 2018. *BMC psychiatry* 2019, 19(1):250. doi: 10.1186/s12888-019-2225-1 31409311 PMC6693166

[35] Sanou AS, Diallo AH, Holding P, Nankabirwa V, Engebretsen IMS, Ndeezi G, et al: Maternal alcohol consumption during pregnancy and child’s cognitive performance at 6–8 years of age in rural Burkina Faso: an observational study. *PeerJ* 2017, 5:e3507. doi: 10.7717/peerj.3507 28674660 PMC5494175

[36] Tafere G: A review on traditional fermented beverages of Ethiopian. *J Nat Sci Res* 2015, 5:94–102.

[37] May PA, Hamrick KJ, Corbin KD, Hasken JM, Marais A-S, Blankenship J, et al: Maternal nutritional status as a contributing factor for the risk of fetal alcohol spectrum disorders. *Reproductive toxicology* 2016, 59:101–108. 26656914 10.1016/j.reprotox.2015.11.006 PMC4783250

[38] Getachew T, Defar A, Teklie H, Gonfa G, Bekele A, Bekele A, et al: Magnitude and predictors of excessive alcohol use in Ethiopia: Findings from the 2015 national non-communicable diseases STEPS survey. *Ethiopian Journal of Health Development* 2017, 31(1):312–319.

[39] Lanting CI, van Dommelen P, van der Pal-de KM, Gravenhorst JB, van Wouwe JP: Prevalence and pattern of alcohol consumption during pregnancy in the Netherlands. *BMC public health* 2015, 15(1):1–5. 26219278 10.1186/s12889-015-2070-1 PMC4517493

[40] Carey KB, Senn TE, Walsh JL, Scott-Sheldon LA, Carey MP: Alcohol use predicts number of sexual partners for female but not male STI clinic patients. *AIDS and Behavior* 2016, 20(1):52–59. 10.1007/s10461-015-1177-9 PMC4715538 26310596

[41] Connery HS, Albright BB, Rodolico JM: Adolescent substance use and unplanned pregnancy: strategies for risk reduction. *Obstetrics and Gynecology Clinics* 2014, 41(2):191–203. doi: 10.1016/j.ogc.2014.02.011 24845484 PMC4031466

[42] Clausen T, Rossow I, Naidoo N, Kowal P: Diverse alcohol drinking patterns in 20 African countries. *Addiction* 2009, 104(7):1147–1154. doi: 10.1111/j.1360-0443.2009.02559.x 19426287

[43] Young B, Lewis S, Katikireddi SV, Bauld L, Stead M, Angus K, et al: Effectiveness of mass media campaigns to reduce alcohol consumption and harm: a systematic review. *Alcohol and alcoholism* 2018, 53(3):302–316. doi: 10.1093/alcalc/agx094 29329359 PMC5913684

[44] Hanson JD, Winberg A, Elliott A: Development of a media campaign on fetal alcohol spectrum disorders for Northern Plains American Indian communities. *Health promotion practice* 2012, 13(6):842–847. 22167361 10.1177/1524839911404232 PMC10955521

[45] Lowe JB, Baxter L, Hirokawa R, Pearce E, Peterson JJ: Description of a Media Campaign About Alcohol Use During Pregnancy. *Journal of Studies on Alcohol and Drugs* 2010, 71(5):739–741. doi: 10.15288/jsad.2010.71.739 20731980

